# Muramyl Dipeptide Enhances Lipopolysaccharide-Induced Osteoclast Formation and Bone Resorption through Increased RANKL Expression in Stromal Cells

**DOI:** 10.1155/2015/132765

**Published:** 2015-04-27

**Authors:** Masahiko Ishida, Hideki Kitaura, Keisuke Kimura, Haruki Sugisawa, Tomo Aonuma, Haruhiko Takada, Teruko Takano-Yamamoto

**Affiliations:** ^1^Division of Orthodontics and Dentofacial Orthopedics, Department of Translational Medicine, Tohoku University Graduate School of Dentistry, 4-1 Seiryo-machi, Aoba-ku, Sendai 980-8575, Japan; ^2^Department of Microbiology and Immunology, Tohoku University Graduate School of Dentistry, 4-1 Seiryo-machi, Aoba-ku, Sendai 980-8575, Japan

## Abstract

Lipopolysaccharide (LPS) is bacterial cell wall component capable of inducing osteoclast formation and pathological bone resorption. Muramyl dipeptide (MDP), the minimal essential structural unit responsible for the immunological activity of peptidoglycans, is ubiquitously expressed by bacterium. In this study, we investigated the effect of MDP in LPS-induced osteoclast formation and bone resorption. LPS was administered with or without MDP into the supracalvariae of mice. The number of osteoclasts, the level of mRNA for cathepsin K and tartrate-resistant acid phosphatase (TRAP), the ratio of the bone destruction area, the level of tartrate-resistant acid phosphatase form 5b (TRACP 5b), and C-terminal telopeptides fragments of type I collagen as a marker of bone resorption in mice administrated both LPS and MDP were higher than those in mice administrated LPS or MDP alone. On the other hand, MDP had no effect on osteoclastogenesis in parathyroid hormone administrated mice. MDP enhanced LPS-induced receptor activator of NF-*κ*B ligand (RANKL) expression and Toll-like receptor 4 (TLR4) expression *in vivo* and in stromal cells *in vitro*. MDP also enhanced LPS-induced mitogen-activated protein kinase (MAPK) signaling, including ERK, p38, and JNK, in stromal cells. These results suggest that MDP might play an important role in pathological bone resorption in bacterial infection diseases.

## 1. Introduction

Lipopolysaccharide (LPS) is a major component of the cell wall of Gram-negative bacteria and is a well-known potent inducer of inflammation and inflammatory bone loss [1–5]. LPS is known to induce the production of many local factors, including proinflammatory cytokines, such as TNF-*α* and IL-1, from macrophages or other cells involved in mediating the inflammatory response in tissues [6]. There is reason to suggest that osteoclast recruitment could be central to diseases involving bone erosion, such as rheumatoid arthritis [7], periprosthetic bone loss [8], postmenopausal osteoporosis, [9] and periodontal disease [2]. Osteoclasts derived from bone marrow cells regulate bone resorption and remodeling [10]. Such osteoclast formation and activation require the expression of two factors: receptor activator of NF-*κ*B ligand (RANKL) and macrophage colony stimulating factor (M-CSF) [11]. Furthermore, tumor necrosis factor- (TNF-) *α* has also been reported to induce osteoclast formation* in vitro* [12–14] and* in vivo* [15, 16]. These inflammatory cytokines have been linked with LPS-induced osteoclast formation and bone destruction* in vivo* and* in vitro* [2, 17–20].

Peptidoglycan (PGN) is another major component of bacterial cell membranes. Muramyl dipeptide (MDP), the minimal essential structural unit responsible for the immunological activity of PGNs, is distributed ubiquitously in the cell walls of both Gram-negative and Gram-positive bacteria. It has been reported that MDP can enhance the production of TNF-*α* when injected into mice [21] and can cause lethal shock in mice challenged with LPS [22]. In addition, MDP has been shown to synergistically enhance LPS-induced proinflammatory cytokine production in human monocyte cells [23]. MDP alone cannot induce osteoclast formation in mouse cocultures of primary osteoblasts and hematopoietic cells; however, it can enhance osteoclast formation induced by LPS, IL-1*α*, and TNF-*α* but not by 1*α*,25-dihydroxy-vitamin-D_3_ (1*α*,25(OH)_2_D_3_) or prostaglandin-E2 (PGE2). Indeed, it has been shown that MDP can upregulate RANKL expression in osteoblasts treated with LPS or TNF-*α* but not those treated with 1*α*,25(OH)_2_D_3_ [24].

In this study, we show that MDP enhances LPS-induced osteoclast formation* in vivo* and increases the expression of RANKL* in vivo* and in stromal cell cultures* in vitro*. MDP also enhances the LPS-induced expression of TLR4—a signal transducing receptor for LPS—both* in vivo* and in stromal cells* in vitro*. Finally, MDP enhances LPS-induced MAPK signaling pathways in stromal cells.

## 2. Material and Methods

### 2.1. Mice and Reagents

Two- to 10-week-old male C57BL6/J mice were purchased from CLEA Japan (Tokyo, Japan) for use in this study. All animal procedures were in accordance with Tohoku University regulations.* Escherichia coli* LPS was purchased from Sigma-Aldrich (St. Louis, MO). MDP (Peptide Institute, Inc., Osaka, Japan) was purchased from Sigma-Aldrich. The following mouse antibodies were obtained from Cell Signaling Technology Inc. (Beverly, MA): polyclonal anti-phospho-p44/42ERK, anti-phospho-JNK, anti-phospho-p38, anti-*β*-Actin, and anti-rabbit IgG horseradish peroxidase- (HRP-) linked antibodies.

### 2.2. Preparation for Histological Observation

Mice calvariae were injected daily for 5 days with PBS, LPS alone (10 *μ*g/day or 100 *μ*g/day, referred to as low or high, resp.), MDP (100 *μ*g/day) alone, or LPS (10 *μ*g/day) and MDP (100 *μ*g/day) (LPS + MDP). The mice were then sacrificed, and the calvariae were immediately harvested and fixed overnight in 4% paraformaldehyde at 4°C. Samples were then demineralized in 14% ethylene-diaminetetraacetic acid for 3 days at 4°C. The sections were stained for TRAP activity and counterstained with hematoxylin for analysis of osteoclast formation. Osteoclasts were counted at the sagittal suture. To cancel out any variations, the calvariae were divided into three sections by the coronal plane. Osteoclasts in five sagittal sutures per section were counted and averaged. In addition, the percentage of interface of bone marrow space covered by osteoclasts was histomorphometrically determined in specimens derived from each sample.

### 2.3. Serum Tartrate-Resistant Acid Phosphatase 5b (TRACP 5b) Assay and Serum C-Terminal Telopeptide Fragments of Type I Collagen Cross-Links (CTX) Assay

Serum was obtained from mice after 5 days of daily LPS administration with or without MDP. The levels of TRACP 5b were determined using a Mouse TRAP Assay kit (IDS, Tyne and Wear, UK). TRACP 5b levels were measured at 405 nm using an absorption microplate reader (model 550; Bio-Rad, Richmond, CA). The levels of C-terminal telopeptide fragments of type I collagen were determined using a Mouse CTX Assay kit (IDS, Tyne and Wear, UK). C-terminal telopeptide fragments of type I collagen levels were measured at 450 nm using an absorption microplate reader (model 550; Bio-Rad, Richmond, CA).

### 2.4. RNA Preparation and Real-Time Reverse-Transcription Polymerase Chain Reaction (RT-PCR) Analysis* In Vitro* and* In Vivo*

For* in vitro* experiments, bone marrow cells from the femora and tibiae of mice were flushed with culture medium. The harvested cells were incubated in Dulbecco's modified Eagle's medium (DMEM; Sigma-Aldrich) containing 10% fetal bovine serum, 100 IU/mL penicillin G (Life Technologies, Carlsbad, CA), and 100 *μ*g/mL streptomycin (Life Technologies). After 2 weeks of culture, cells were washed with PBS to remove floating cells. Adherent cells from these cultures were used as bone marrow stromal cells in this study. Adherent bone marrow stromal cells were incubated in culture medium supplemented with high or low LPS alone, LPS + MDP, or MDP alone. After 3 days of culture, total RNA was isolated from adherent cells using an RNeasy mini kit (Qiagen, Valencia, CA).

For* in vivo* experiments, harvested calvariae were frozen in liquid nitrogen, ground using a Micro Smash MS-100R (TOMY SEIKO, Tokyo, Japan), and then centrifuged in 800 *μ*L of TRIzol Reagent (Invitrogen, Carlsbad, CA). RNA was isolated from these samples using an RNeasy minikit (Qiagen). All cDNA was synthesized from 2 *μ*g of total RNA using reverse transcriptase and oligo-dT primers (Invitrogen) in a reaction volume of 20 *μ*L. The mRNA expression levels of TRAP, cathepsin K, RANKL, and TLR4 were quantified by real-time RT-PCR using a Thermal Cycler Dice Real Time System (Takara, Shiga, Japan). Reactions were performed in a 25 *μ*L volume containing 2 *μ*L of cDNA, 12.5 *μ*L of SYBR Premix Ex Taq (Takara), and 25 pmol/*μ*L primers. The cycling conditions were as follows: 95°C for 10 s for initial denaturation followed by 45 cycles of amplification, with each cycle consisting of a denaturation step at 95°C for 5 s and an annealing step at 60°C for 30 s. Gene expression levels were normalized to glyceraldehyde 3-phosphate dehydrogenase (GAPDH) mRNA. The following primers were used: for GAPDH, 5′-GGTGGAGCCAAAAGGGTCA-3′ and 5′-GGGGGCTAAGCAGTTG-GT-3′; cathepsin K, 5′-GCAGAGGTTGTACTATGA-3′ and 5′-GCAGGCGTTGTTCTTATT-3′; TRAP, 5′-AACTTGCGACCATTGTTA-3′ and 5′-GGGGACCTTTCGTTGATGT-3′; RANKL, 5′-CCTGAGGCCAGCCATTT-3′ and 5′-CTTGGCCCAGCCT-3′; and TLR4, 5′-CACTGTTCTTCTCCTGCCTGAC-3′ and 5′-TGGTTGAAGAAGGAATGTCATC-3′.

### 2.5. Measurement of Bone Destruction

Calvariae were harvested and the soft tissues were carefully removed. Calvariae were then fixed in PBS-buffered formaldehyde (4%) for 3 days at 4°C and then washed with PBS for radiological analysis. Microfocus computed tomography (ScanXmate-E090; Comscan, Kanagawa, Japan) was used to assay the bone resorption pits in the calvariae, and TRI/3D-BON64 software (RATOC System Engineering, Tokyo, Japan) was used to build three-dimensional reconstruction images of the calvariae. The ratio of bone destruction to total area was calculated using ImageJ (NIH, Bethesda, MD).

### 2.6. Immunoblotting for Analysis of MAPK Signaling

Stromal cells were cultured in serum-free DMEM for 3 h before treatment with LPS and/or MDP for the various durations, as indicated. Treated cells were washed twice with ice-cold PBS and then lysed in lysis buffer (Cell Signaling Technology) containing a protease inhibitor mixture. Cell lysates (30 *μ*g) were boiled in the presence of lithium dodecyl sulfate sample buffer (Life Technologies) for 5 min and subjected to SDS polyacrylamide gel electrophoresis using 4–15% Mini-PROTEAN TGX gels (Bio-Rad, Hercules, CA). Proteins were transferred to nitrocellulose membranes using Trans-Blot Turbo (Bio-Rad) and incubated in blocking solution (5% bovine serum albumin in Tris-buffered saline containing 0.05% Tween-20) for 1 h to reduce nonspecific binding. Membranes were then exposed to primary antibodies for 1 h at 4°C, washed four times, and then incubated with anti-rabbit IgG HRP-conjugated secondary antibody for 30 min. Membranes were again washed extensively and then incubated with enhanced chemiluminescence detection using Supersignal West Femto Maximum Sensitivity Substrate (Thermo Fisher Scientific, Wilmington, DE).

### 2.7. Statistical Analysis

All data are expressed as the mean ± SD. Statistical analyses were performed using Scheffe's *F* test.

## 3. Results

### 3.1. MDP Enhances LPS-Induced Osteoclastogenesis in Mouse Calvariae

**Figure 1:**
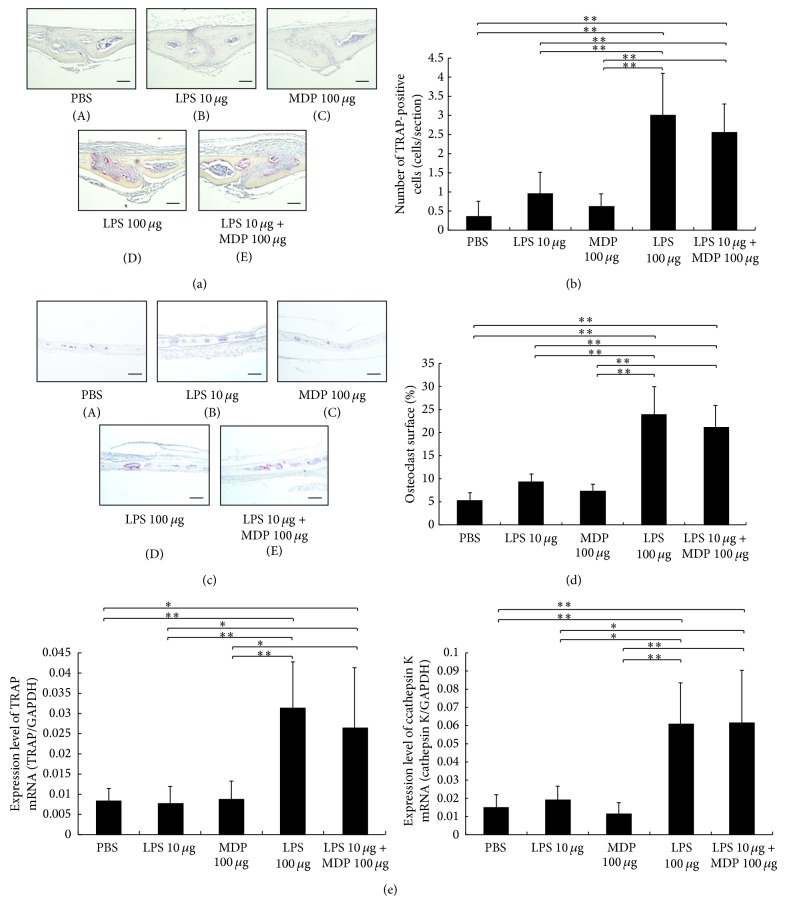
Effects of muramyl dipeptide (MDP) on lipopolysaccharide- (LPS-) induced osteoclast formation* in vivo*. (a) Histological sections of sutures of calvariae were obtained from mice after 5 days of daily supracalvarial injections of one of the following: PBS (A), 10 *μ*g/day LPS (B), 100 *μ*g/day MDP (C), 100 *μ*g/day LPS (D), or 10 *μ*g/day LPS + 100 *μ*g/day MDP (E). Sections were stained with tartrate-resistant acid phosphatase (TRAP) staining and counterstained with hematoxylin. Cells that stained red are considered to be TRAP-positive. Scale bars = 50 *μ*m. (b) The number of TRAP-positive cells with three or more nuclei in the calvariae (*n* = 4; ^**^ *P* < 0.01). (c) Histological sections of calvariae were obtained from mice after 5 days of daily supracalvarial injections of one of the following: PBS (A), 10 *μ*g/day LPS (B), 100 *μ*g/day MDP (C), 100 *μ*g/day LPS (D), or 10 *μ*g/day LPS + 100 *μ*g/day MDP (E). Scale bars = 100 *μ*m. (d) The percentage of bone/marrow interface covered by osteoclasts was histomorphometrically determined in specimens (*n* = 4; ^**^ *P* < 0.01). (e) TRAP and cathepsin K mRNA levels in mouse calvariae detected using real-time RT-PCR. Total RNA from mouse calvariae was isolated after 5 days of daily supracalvarial injections of PBS, LPS (10 *μ*g/day), MDP (100 *μ*g/day), LPS (100 *μ*g/day), or LPS (10 *μ*g/day) + MDP (100 *μ*g/day). mRNA levels for TRAP and cathepsin K were normalized to GAPDH. Results are expressed as the mean ± SD (*n* = 4; ^**^ *P* < 0.01; ^*^ *P* < 0.05). Differences were determined using Scheffe's *F* test.

LPS was administered with or without MDP into the supracalvariae of mice to analyze the effect of MDP on LPS-induced osteoclastogenesis* in vivo*. In the high LPS (100 *μ*g/day) group and the LPS + MDP group, numerous osteoclasts were observed. In comparison, significantly fewer osteoclasts were observed in the low LPS (10 *μ*g/day), MDP alone, or PBS groups (Figures 1(a), 1(b), 1(c), and 1(d)).

Real-time RT-PCR was undertaken to analyze cathepsin K and TRAP mRNA levels—two markers of osteoclasts. We found that both cathepsin K and TRAP mRNA were significantly higher in the LPS + MDP group and the high LPS group as compared with the low LPS group (Figure 1(e)).

### 3.2. Concentration-Dependent Increase in Osteoclastogenesis

**Figure 2:**
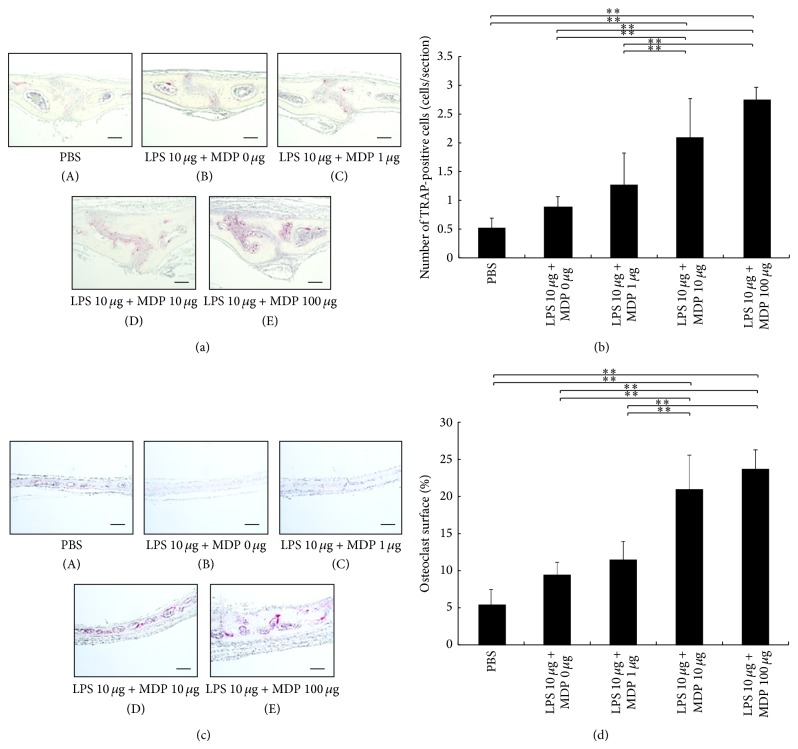
Osteoclast formation is dependent on muramyl dipeptide (MDP) concentration. (a) Osteoclast number in the sutures following treatment with a low concentration of lipopolysaccharide (LPS) (10 *μ*g/day) with increasing concentrations of MDP for 5 days. Sections were stained with tartrate-resistant acid phosphatase (TRAP) staining and counterstained with hematoxylin. Cells that stained red are considered to be TRAP-positive. Scale bars = 50 *μ*m. (b) Number of TRAP-positive cells with three or more nuclei in the calvariae (*n* = 4; ^**^ *P* < 0.01). (c) Osteoclast number in the bone/marrow interface following treatment with a low concentration of lipopolysaccharide (LPS) (10 *μ*g/day) with increasing concentrations of MDP for 5 days. Sections were stained with tartrate-resistant acid phosphatase (TRAP) staining and counterstained with hematoxylin. Cells that stained red are considered to be TRAP-positive. Scale bars = 100 *μ*m. (d) The percentage of bone/marrow interface covered by osteoclasts was histomorphometrically determined in specimens (*n* = 4; ^**^ *P* < 0.01). Differences were detected using Scheffe's *F* test.

To further analyze the effect of MDP on LPS-induced osteoclast formation* in vivo*, LPS (10 *μ*g/day) was injected into mouse calvariae with increasing concentrations of MDP (0, 1, 10, and 100 *μ*g). We found that higher MDP concentrations led to an increase in osteoclast number in a dose-dependent manner (Figure 2).

### 3.3. MDP Enhances LPS-Induced Bone Destruction in Supracalvariae

**Figure 3:**
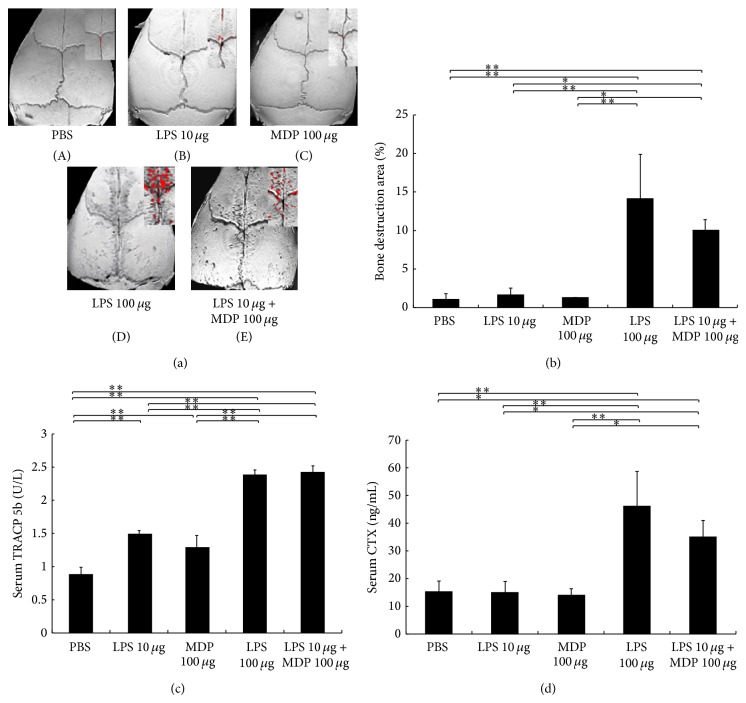
Muramyl dipeptide (MDP) enhances lipopolysaccharide- (LPS-) induced bone destruction in mouse calvariae. (a) Microfocus computed tomography reconstruction images of mouse calvariae harvested after 5 days of daily administration of PBS (A), 10 *μ*g/day LPS (B), 100 *μ*g/day MDP (C), 100 *μ*g/day LPS (D), or 10 *μ*g/day LPS + 100 *μ*g/day MDP (E). Red areas indicate larger areas of bone destruction. (b) Ratio of bone destruction area to total area. Results are expressed as the mean ± SD (*n* = 4; ^**^ *P* < 0.01; ^*^ *P* < 0.05). Differences were determined using Scheffe's *F* test. (c) Levels of TRACP 5b in mouse serum* in vivo*. Serum was obtained from mice after five days of daily administration into the calvariae. Circulating levels of tartrate-resistant acid phosphatase (TRACP 5b) were determined by enzyme-linked immunosorbent assay (ELISA). Results are expressed as the mean ± SD (*n* = 4; ^**^ *P* < 0.01). (d) Levels of C-terminal telopeptide fragments of type I collagen in mouse serum* in vivo*. Circulating levels of C-terminal telopeptide fragments of type I collagen were determined by Mouse CTX Assay kit. Results are expressed as the mean ± SD (*n* = 4; ^**^ *P* < 0.01; ^*^ *P* < 0.05). Differences were determined using Scheffe's *F* test.

We next used microfocus computed tomography to assess the degree of bone destruction observed in the calvariae of mice administered with LPS (Figure 3(a)). We found significantly more bone destruction in the high LPS group as compared with the PBS group. In addition, bone destruction in the LPS + MDP group was higher than that in the low LPS group (Figure 3(b)). This increased bone destruction was corroborated by the TRACP 5b serum analysis, where we found that TRACP 5b was increased in the high LPS group as compared with that in the PBS, low LPS, and MDP only groups. Moreover, TRACP 5b serum levels were higher in the LPS + MDP group than in the PBS, low LPS, and MDP only groups (Figure 3(c)). C-terminal telopeptide fragments of type I collagen serum levels were also higher in the LPS + MDP group than in the PBS, low LPS, and MDP only groups (Figure 3(d)).

### 3.4. MDP Enhances LPS-Induced RANKL Expression* In Vivo*

**Figure 4:**
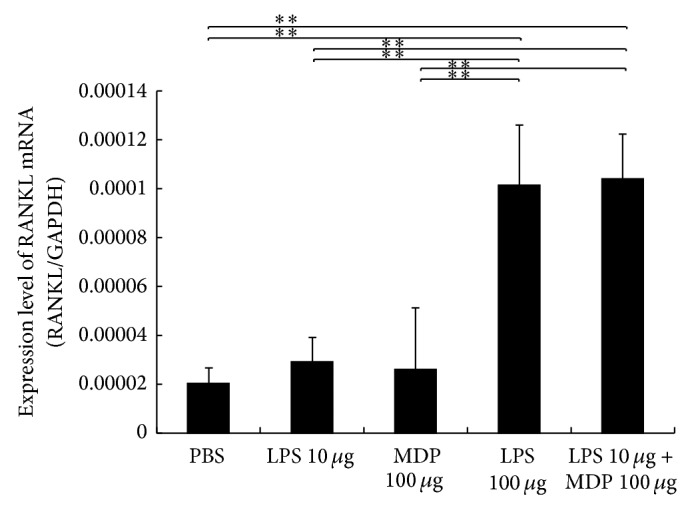
Enhancing lipopolysaccharide- (LPS-) induced receptor activator of NF-*κ*B ligand (RANKL) expression by muramyl dipeptide (MDP)* in vivo*. Total RNA from mouse calvariae was isolated after 5 days of daily supracalvarial injections of PBS, LPS (10 *μ*g/day), MDP (100 *μ*g/day), LPS (100 *μ*g/day), or LPS (10 *μ*g/day) + MDP (100 *μ*g/day). mRNA levels for RANKL were normalized to those of GAPDH. Results are expressed as mean ± SD (*n* = 4; ^**^ *P* < 0.01). Differences were determined using Scheffe's *F* test.

RANKL was related to the osteoclast formation. We found that RANKL mRNA was elevated in the high LPS and LPS + MDP groups as compared with PBS, low LPS, and MDP alone groups. MDP was thus able to enhance LPS-induced RANKL expression* in vivo* (Figure 4).

### 3.5. MDP Enhances LPS-Induced RANKL Expression in Stromal Cells

**Figure 5:**
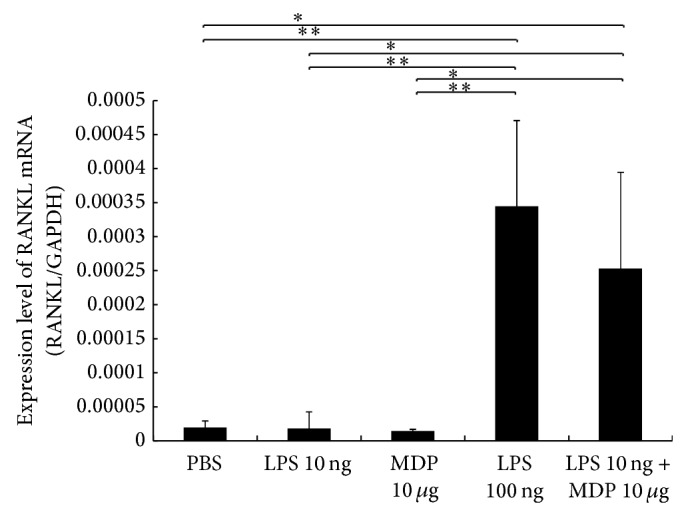
Enhancing lipopolysaccharide- (LPS-) induced receptor activator of NF-*κ*B ligand (RANKL) expression by muramyl dipeptide (MDP) in stromal cells* in vitro*. Total RNA from bone marrow stromal cells was isolated after 4 days of incubation in culture medium supplemented with 10 ng/mL LPS, 100 ng/mL LPS, or 10 ng/mL LPS and 10 *μ*g/mL MDP. mRNA levels for RANKL were normalized to those of GAPDH. Results are expressed as mean ± SD (*n* = 4; ^**^ *P* < 0.01; ^*^ *P* < 0.05). Differences were determined using Scheffe's *F* test.

Bone marrow stromal cells were cultured for 3 days in the presence of LPS with or without MDP to ascertain the effect of these two additives on RANKL expression in stromal cell cultures* in vitro*. We found elevated RANKL mRNA expression in the high LPS group as compared with the PBS, low LPS, and MDP alone groups. Similarly, RANKL mRNA was significantly higher in the LPS + MDP group as compared with the PBS and low LPS groups (Figure 5).

### 3.6. Effect of MDP on Parathyroid Hormone- (PTH-) Induced Osteoclastogenesis in Mouse Calvariae

**Figure 6:**
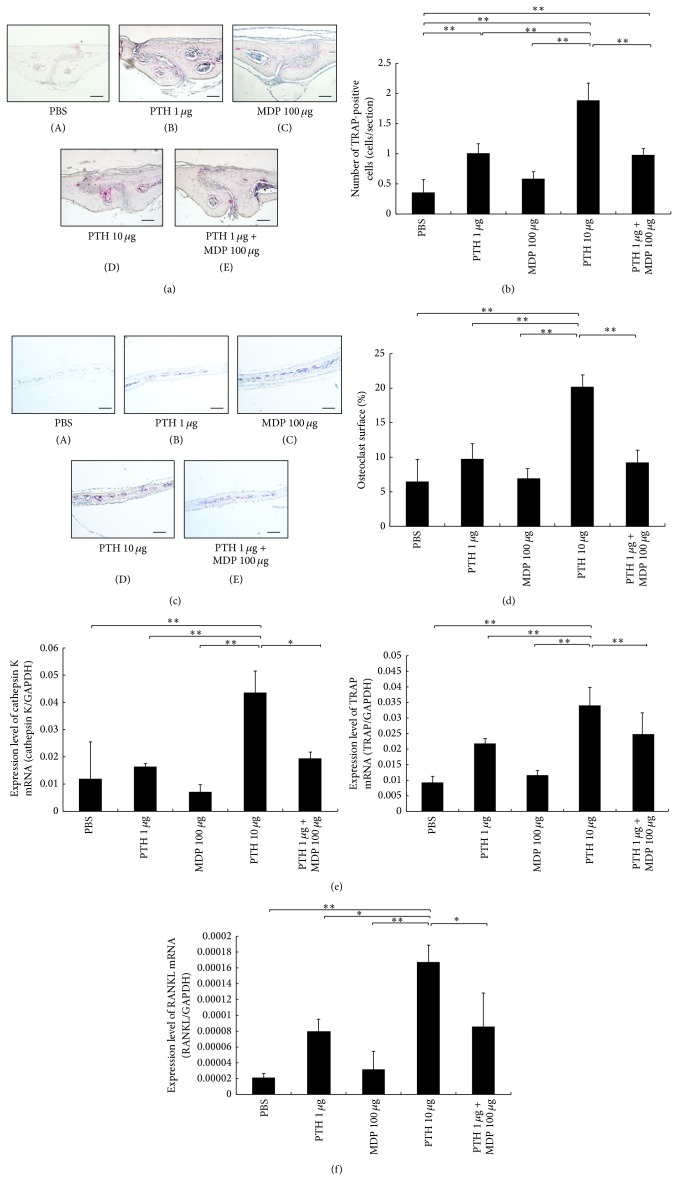
Effects of muramyl dipeptide (MDP) on parathyroid hormone- (PTH-) induced osteoclast formation* in vivo*. (a) Histological sections of sutures of calvariae were obtained from mice after 5 days of daily supracalvarial administration with PBS (A), 1 *μ*g/day PTH (B), 100 *μ*g/day MDP (C), 10 *μ*g/day PTH (D), or 1 *μ*g/day PTH and 100 *μ*g/day MDP (E). Sections were stained with tartrate-resistant acid phosphatase (TRAP) staining and counterstained with hematoxylin. Cells that stained red are considered to be TRAP-positive. Scale bars = 50 *μ*m. (b) Number of TRAP-positive cells with three or more nuclei in the calvariae (*n* = 4; ^**^ *P* < 0.01). (c) Histological sections of calvariae were obtained from mice after 5 days of daily supracalvarial administration with PBS (A), 1 *μ*g/day PTH (B), 100 *μ*g/day MDP (C), 10 *μ*g/day PTH (D), or 1 *μ*g/day PTH and 100 *μ*g/day MDP (E). Sections were stained with tartrate-resistant acid phosphatase (TRAP) staining and counterstained with hematoxylin. Cells that stained red are considered to be TRAP-positive. Scale bars = 100 *μ*m. (d) The percentage of bone/marrow interface covered by osteoclasts was histomorphometrically determined in specimens (*n* = 4; ^**^ *P* < 0.01). (e) TRAP and cathepsin K mRNA levels in mouse calvariae were detected using real-time RT-PCR. Total RNA from mouse calvariae was isolated after 5 days of daily supracalvarial injections, as in (a). RNA levels for TRAP and cathepsin K were normalized to those of GAPDH. Results are expressed as the mean ± SD (*n* = 4; ^**^ *P* < 0.01; ^*^ *P* < 0.05). (f) Expression levels of RANKL mRNA in mouse calvariae* in vivo*. Total RNA from mouse calvariae was isolated after 5 days of daily supracalvarial injections, as in (a). mRNA levels for RANKL were normalized to those of GAPDH. Results are expressed as the mean ± SD (*n* = 4; ^**^ *P* < 0.01; ^*^ *P* < 0.05). Differences were determined using Scheffe's *F* test.

PTH stimulates RANKL expression by osteoblasts and thus indirectly stimulates osteoclastogenesis. We therefore sought to ascertain if PTH could similarly be enhanced by MDP. PTH (100 *μ*g/day) was administered with or without MDP into mouse supracalvaria to analyze the effect of MDP on PTH-induced osteoclastogenesis* in vivo*. We observed numerous osteoclasts with the higher concentration of PTH (10 *μ*g/day), which was significantly diminished in mice treated with low-dose PTH (1 *μ*g/day), PTH (1 *μ*g/day) + MDP, MDP alone, or PBS (Figures 6(a), 6(b), 6(c), and 6(d)). Cathepsin K and TRAP mRNA levels were significantly increased in the high PTH group as compared with the PTH + MDP, PBS, low PTH, and MDP alone groups (Figure 6(e)).

### 3.7. Effect of MDP on RANKL Expression in PTH-Administered Mice

Mice calvariae were injected daily for 5 days with PTH (1 *μ*g) + MDP (100 *μ*g) in a 100 *μ*L volume of PBS or separately with high PTH (10 *μ*g), low PTH (1 *μ*g), MDP (100 *μ*g), or PBS alone to ascertain the effect of these compounds on RANKL. We found that RANKL mRNA was higher in the high PTH group than in the PTH + MDP, PBS, low PTH, or MDP alone groups (Figure 6(f)).

### 3.8. MDP Enhanced LPS-Induced TLR4 Expression* In Vivo*

**Figure 7:**
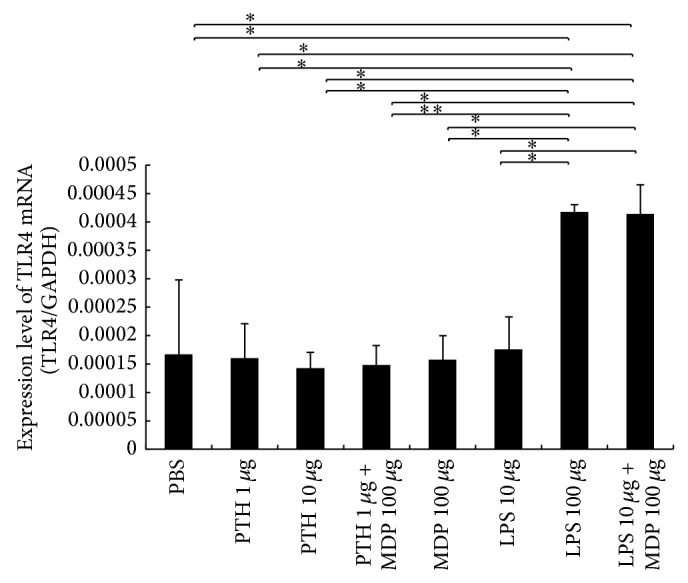
Enhancing lipopolysaccharide- (LPS-) induced Toll-like receptor (TLR4) expression using muramyl dipeptide (MDP)* in vivo*. Total RNA from mouse calvariae was isolated after 5 days of daily supracalvarial injections of PBS, PTH (1 *μ*g/day), PTH (10 *μ*g/day), PTH (1 *μ*g/day) + MDP (100 *μ*g/day), MDP (100 *μ*g/day), LPS (10 *μ*g/day), LPS (100 *μ*g/day), or LPS (10 *μ*g/day) + MDP (100 *μ*g/day). mRNA levels for TLR4 were normalized to those of GAPDH. Results are expressed as the mean ± SD (*n* = 4; ^**^ *P* < 0.01; ^*^ *P* < 0.05). Differences were determined using Scheffe's *F* test.

We next determined the effect of MDP on LPS- and PTH-induced TLR4 expression, a receptor for LPS. We found that TLR4 mRNA expression levels were higher in the high LPS and LPS + MDP groups than in the PBS, low LPS, and MDP alone groups. On the other hand, PTH did not induce TLR4 mRNA and MDP did not enhance TLR4 mRNA in the presence of PTH (Figure 7).

### 3.9. MDP Enhanced LPS-Induced TLR4 Expression in Stromal Cells

**Figure 8:**
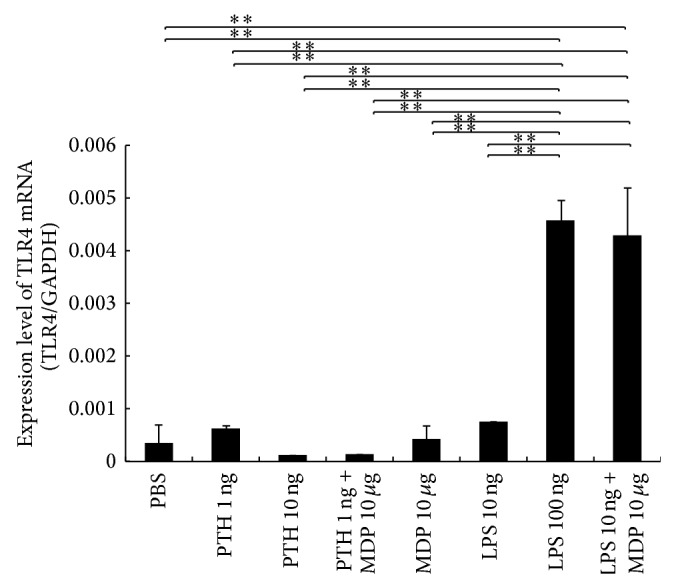
Enhancing lipopolysaccharide- (LPS-) induced Toll-like receptor (TLR4) expression using muramyl dipeptide (MDP)* in vitro*. Total RNA from bone marrow stromal cells was isolated after 4 days of incubation in culture medium supplemented with 1 ng/mL PTH, 10 ng/mL PTH, 1 ng/mL PTH and 10 *μ*g/mL MDP, 10 *μ*g/mL MDP, 100 ng/mL LPS, or 10 ng/mL LPS + 10 *μ*g/mL MDP. mRNA levels for TLR4 were normalized to those of GAPDH. Results are expressed as the mean ± SD (*n* = 4; ^**^ *P* < 0.01). Differences were determined using Scheffe's *F* test.

Bone marrow stromal cells were cultured for 3 days in LPS or PTH with or without MDP. In these cultures, we show that TLR4 mRNA with high LPS (100 ng/mL) was higher than that in the PBS, low LPS (10 ng/mL), or MDP alone groups. In addition, TLR4 mRNA expression in the LPS (10 ng/mL) + MDP group was significantly higher than that in the PBS and low LPS (10 ng/mL) groups. As seen in the* in vivo* analysis, PTH was also unable to induce TLR4 mRNA in stromal cells and this could not be recovered with the coadministration of MDP (Figure 8).

### 3.10. MDP Enhanced LPS-Induced MAPK Signaling Pathway in Stromal Cells

**Figure 9:**
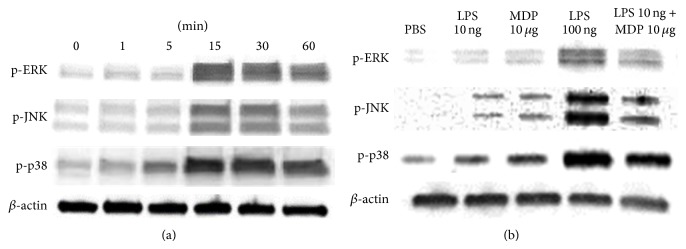
Effect of muramyl dipeptide (MDP) on lipopolysaccharide- (LPS-) induced mitogen-activated protein kinase (MAPK) signaling in mouse stromal cell* in vitro*. (a) Stromal cells were stimulated using LPS (100 ng/mL) as indicated. Cells were then lysed and analyzed by western blotting. (b) Stromal cells were stimulated using PBS, LPS (10 ng/mL), MDP (10 *μ*g/mL), LPS (100 ng/mL), or LPS (10 ng/mL) + MDP (10 *μ*g/mL) for 15 min. Cells were then lysed and analyzed by western blotting.

Finally, we sought to explore the molecular mechanisms through which MDP enhances LPS-activated signaling. We showed that LPS activated ERK, P38, and JNK in mouse bone marrow stromal cells after 15 min incubation. MDP alone was unable to induce phosphorylation of any of the kinases; however, MDP enhanced LPS-induced phosphorylation of all three kinases after just 15 min of incubation (Figure 9).

## 4. Discussion

In this study, we evaluated the effect of MDP in LPS-induced osteoclast formation and bone resorption* in vivo*. To our knowledge, this is the first time that this analysis has been reported. We found that MDP enhances LPS-induced osteoclast formation and bone resorption and also enhances LPS-induced RANKL and TLR4 expression* in vivo* and in stromal cell* in vitro*. Furthermore, MDP enhanced LPS-induced phosphorylation of ERK, p38, and JNK kinases in stromal cells, although MDP alone could not induce their activity.

It has been reported that LPS can induce osteoclast formation and bone resorption in certain clinical conditions, such as periodontal diseases [2, 25]. We have previously shown that osteoclasts can be induced in calvariae [26] and in periodontal membrane tissues [27] in the presence of LPS. Yang et al. [24] showed that MDP enhances LPS-induced osteoclast formation when cocultured with osteoblasts* in vitro*. In the present study, we evaluated whether MDP could enhance LPS-induced osteoclast formation and bone resorption* in vivo*. First, we analyzed the amount of LPS required for osteoclast formation. We found that a daily injection of 100 *μ*g/day for 5 days was sufficient to induce osteoclasts* in vivo*, but not with injections of 10 *μ*g/day for 5 days. Next, to analyze the effect of MDP on LPS-induced osteoclastogenesis* in vivo*, the lower concentration of LPS was administered with or without MDP into mouse supracalvaria. We found increased numbers of osteoclasts and an elevated expression of osteoclast markers (cathepsin K and TRAP) with high LPS (100 *μ*g/day) and with low LPS (10 *μ*g/day) plus MDP but not with low LPS (10 *μ*g/day) or MDP alone or with the vehicle, PBS. These results suggest that MDP can enhance LPS-induced osteoclast formation* in vivo*.

Furthermore, we evaluated whether MDP could enhance LPS-induced bone resorption. Bone destruction was observed using microfocus computed tomography images. Serum TRACP 5b levels with LPS (10 *μ*g) plus MDP were higher than that in the LPS only group. These results suggest that MDP enhances LPS-induced bone resorption. Kishimoto et al. [28] investigated the effect of PGN on LPS-induced osteoclast formation and bone resorption and found that PGN significantly induced osteoclast formation and bone resorption in mice coinjected with LPS. MDP is the minimal essential structural unit responsible for the immunological activity of PGN [29]. Thus, it is likely that MDP might be the key component in LPS-induced osteoclast formation and bone resorption as mediated by PGN.

LPS has also been reported to stimulate osteoblast production/secretion of RANKL [30]. In the present study, we, too, found elevated RANKL mRNA levels in the high-dose LPS group as compared with the control groups both* in vivo* and* in vitro*, indicating that LPS induced RANKL expression in stromal cells. Yang et al. also examined osteoblasts cultured in the presence of LPS with or without MDP. They showed that MDP stimulated the LPS-induced expression of RANKL mRNA [24]. Our results with stromal cells support these previous findings. However, we showed that MDP alone could not induce RANKL expression either* in vitro* or* in vivo*, suggesting that MDP enhances the effect of LPS.

PTH stimulates RANKL expression by osteoblasts and thus promotes osteoclastogenesis [31–35]. We also evaluated whether MDP could enhance PTH-induced osteoclast formation and bone resorption. In the present study, PTH induced osteoclast formation and bone resorption in mouse calvariae. However, MDP could not enhance PTH-induced osteoclast formation and bone resorption. The results suggested that although MDP affects LPS-induced signaling it cannot affect PTH-induced signaling.

TLR4 induces the natural host defense system by rapidly triggering proinflammatory processes [36–38]. LPS is recognized by TLR4 on the cell surface [39, 40]. In this study, we found that LPS enhances TLR4 expression in mouse calvariae and in stromal cell culture. PTH, however, could not enhance TLR4 expression. We hypothesize that this phenomenon might increase the sensitivity of LPS in cells. Furthermore, we found that MDP could enhance LPS-induced TLR4 expression* in vivo* and in stromal cells. These results provide further support for the premise that MDP enhances LPS signaling, and its signaling through TLR4 may be how MDP enhances the effects of LPS.

Cyclooxygenase- (COX-) 2 and PGE2 are reportedly increased in dental pulp fibroblasts by costimulation with NOD1 or NOD2 ligands and TLR2 or TLR4 ligands. Furthermore, the production of IL-1*β*, IL-6, and IL-8 in these fibroblasts is accelerated by costimulation with these ligand combinations through the increased expression of TRAF6 [41]. It has been reported that MDP synergistically enhances osteoclast induction by LPS, IL-1*α*, and TNF-*α* through increased RANKL expression in osteoblasts [24]. Bandow et al. [42] and Nakao et al. [43] have also shown that LPS activates the phosphorylation of ERK, P38, and JNK in osteoblasts. We corroborated these results, showing that LPS activates all three kinases in mouse bone marrow stromal cells. Yang et al. [24] also showed that LPS stimulated ERK1/2 phosphorylation in osteoblasts and that this could be enhanced by MDP. However, they did not check the effect of LPS on other MAPKs, such as p38 and JNK. We found that MDP enhanced the phosphorylation of ERK, p38, and JNK that was induced by LPS in stromal cells. Yet, MDP alone was unable to activate MAPKs. Although these results provide some insight into the signaling pathways activated by LPS, the exact mechanism by which MDP enhances LPS signaling is unclear, and further studies are needed to clarify this point.

## 5. Conclusions

We found that MDP enhances LPS-induced osteoclast formation, as measured by increased RANKL and TLR4 expression* in vivo* and* in vitro*. Our findings suggest that MDP might play an important role in pathological bone resorption in diseases with associated bacterial infections, such as periodontitis.
